# Comment on “Multiomics Analysis Reveals Therapeutic Targets for Chronic Kidney Disease With Sarcopenia” by Wang et al.

**DOI:** 10.1002/jcsm.70073

**Published:** 2025-09-23

**Authors:** Cedric Moro

**Affiliations:** ^1^ Institute of Metabolic and Cardiovascular Diseases (I2MC) Inserm/Toulouse University UMR1297 Toulouse France

1

In a recent issue of *J Cachexia Sarcopenia Muscle*, Wang and colleagues [1] investigated the influence of renal secretions on muscle wasting in the context of chronic kidney diseases (CKD), taking advantage of multi‐omics profiling of kidneys, serum and skeletal muscle. CKD is a global public health concern that affects approximately 10%–15% of people worldwide. CKD represents a state of hyperuricemia with progressive and irreversible loss of kidney function, and its prevalence increases with age, particularly in individuals with hypertension and diabetes [2]. CKD is frequently associated with severe loss of muscle mass and force that negatively impacts the quality of life of patients, leading to higher risks of frailty, co‐morbidities and mortality [3]. Taking advantage of various mouse models of CKD, several molecular mechanisms of cachexia and muscle wasting have been reported [4, 5, 6]. The 5/6 nephrectomy (Nx) model, which involves surgical resection of kidney mass, is one of the most widely used techniques to successfully induce renal failure in laboratory animals [7]. Another model is the adenine diet model of CKD, a nonsurgical option, that was developed to induce renal damages in rodents. Kidney disease with adenine feeding stems from the formation of 2,8‐dihydroxyadenine, an adenine metabolite that crystalizes within renal tubules and causes injury, inflammation, tubular atrophy and fibrosis of the renal parenchyma. Interestingly, Nx and adenine models seem to produce equivalent levels of nephropathy and muscle atrophy [7].

In their study, Wang and colleague [1] used the adenine model to induce CKD by feeding 8‐week‐old male C57BL/6JNifdc mice a 0.2% supplemented adenine diet for 6 weeks. As expected, blood urea nitrogen and serum creatinine levels were markedly increased in adenine diet‐fed mice, while mice experienced a dramatic weight loss of ~40% within 6 weeks as well as a major loss of muscle mass and force (~45%). Next, they performed bulk RNA‐sequencing and proteomics of kidneys and *gastrocnemius* muscles to identify a few up‐regulated proteins such as Secreted Phosphoprotein 1 (SPP1) also known as osteopontin and S100 Calcium Binding Protein A9 (S100A9) in CKD mice. They further speculate about the potential pro‐atrophic effects of these two proteins using cultured C2C12 mouse myotubes.

However, we recently demonstrated that one major caveat of the adenine diet CKD mouse model is the major appetite suppression induced by the adenine diet reaching 60%–70% and the complete lack of correlation between kidney dysfunction and muscle wasting [8]. We estimated daily food consumption in the adenine diet to be on average 1.5 g/day, which corresponds to 2–3 times less than what control mice usually eat (3.5–4 g/day). This is essentially due to the poor palatability of adenine‐enriched diets [7, 9]. We further showed that lean mass, muscle mass, muscle force and cross‐sectional area were fully recovered 6 weeks after discontinuing the adenine diet despite major kidney failure, with a severe drop in glomerular filtration rate, kidney fibrosis and elevated plasma creatinine and urea. Importantly, we pushed the investigation up to 25 weeks after removal of the adenine diet and found similar results with a remarkably preserved muscle mass and function [8].

In summary, our data indicate that caution should be taken when interpreting changes in body composition, muscle mass and function in preclinical CKD mouse models. Body weight and composition changes in the adenine‐induced nephropathy model are largely mediated by a dramatic energy deficit and are unrelated to kidney dysfunction. Thus, mice can fully recover their initial lean body mass, and no obvious signs of cachexia or muscle wasting are observed after discontinuing the adenine diet. Although this model may be suitable to induce CKD in mice, it does not seem appropriate to investigate mechanisms and therapeutic strategies of CKD‐related cachexia. Therefore, although we generally agree that CKD could be associated with transcriptome and proteome changes in kidneys and muscles induced by uremic toxins and other factors, we strongly urge the scientific community to consider this caveat in future studies to increase clinical translation of their findings.

## Conflicts of Interest

The author declares no conflicts of interest.
